# Complete Genome Sequence of an Emerging Melon Necrotic Spot Virus Isolate Infecting Greenhouse Cucumber in North America

**DOI:** 10.1128/genomeA.00775-15

**Published:** 2015-07-16

**Authors:** Rugang Li, Yi Zheng, Zhangjun Fei, Kai-Shu Ling

**Affiliations:** a U.S. Department of Agriculture, Agricultural Research Service, U.S. Vegetable Laboratory, Charleston, South Carolina, USA; b Boyce Thompson Institute for Plant Research, Cornell University, Ithaca, New York, USA; c Robert W. Holley Center for Agriculture and Health, USDA, Agricultural Research Service, Ithaca, New York, USA

## Abstract

The complete genome sequence (4,267 nt) of a *Melon necrotic spot virus* (MNSV) isolate (ABCA13-01) infecting greenhouse cucumber in Canada was determined through deep sequencing of small RNAs. Its genome sequence was most closely related to MNSV-N (97%) but lacked a 55-nucleotide insertion at the 3′ untranslated region for resistance breaking.

## GENOME ANNOUNCEMENT

*Melon necrotic spot virus* (MNSV), a small isometric virus (30-nm diameter), belongs to the genus *Carmovirus* in the family *Tombusviridae* (1). MNSV is naturally transmitted by the soil-inhabiting fungus *Olpidium radicale* (2, 3) and by seeds (4, 5). It can also be transmissible by mechanical inoculation. MNSV infects mainly cucurbits, including melon, cucumber, and watermelon in the field, causing serious yield loss (6). The virus is widely distributed in Asia, Europe, and North America, including Japan (4), the United States (5), Greece (7), Sweden (8), Italy (9), The Netherlands (10, 11), Tunisia (12), China (13), and Spain (14, 15). 

MNSV genomic RNA is about 4.3 kb in size (11) and contains four open reading frames (ORFs). Translation of an MNSV genome yields a polypeptide of 29 kDa (p29) and a read-through polypeptide of 89 kDa (p89) in ORF1. ORFs 2 and 3 code for two small polypeptides, each of about 7 kDa (p7A and p7B), which are involved in virus cell-to-cell movement. ORF4 codes for the 42-kDa coat protein (p42). Currently, a total of 18 complete or near-complete MNSV genome sequences are available in GenBank. From a viral disease outbreak on greenhouse cucumber in 2013 in Alberta, Canada, in addition to the *Cucumber green mottle mosaic virus* previously identified (16, 17), 8 of the 10 samples analyzed also contained a mixed infection of MNSV. Upon mechanical inoculation, necrotic spots were observed on the inoculated plants in several cucurbit species, including *Cucumis sativus, C. metulifer*, *C. melo*, and *Citrullus lanatus* and confirmed for the presence of MNSV by real-time RT-PCR (R. Li and K.-S. Ling, unpublished data). MNSV was biologically purified through three local lesion passages on *C. metulifer*. Symptomatic cotyledons were collected for total RNA isolation using the TRIzol reagent (Invitrogen, USA). The small RNA (sRNA) was separated from the total RNA by polyacrylamide gel electrophoresis, and the sRNA library was constructed according to the published protocol (18) and sequenced on an Illumina HiSeq 2000 system. The sRNA sequences were assembled into contigs using a bioinformatics pipeline (19). A single contig (4,267 nt) covering a complete MNSV genome was obtained and deposited in GenBank (no. KR094068). A BLASTn search using the full-genome sequence indicated that this Canadian isolate (ABCA13-01) shares a genome sequence identity of 80% to 97% to the other 18 MNSV isolates. Interestingly, the most closely related isolate is MNSV-N (KF060715), a new virulent isolate in Spain that breaks the host resistance (15). 

Although they shared the strongest sequence identity, the 55-nucleotide insertion in the 3′ untranslated region of MNSV-N that is responsible for the resistance breaking (15) was not present in isolate ABCA13-01. The close phylogenetic relationship to the European isolates of MNSV suggests that this Canadian isolate might have been introduced in seeds from Europe. To our knowledge, this is the first complete genome sequence of MNSV in Canada.

### Nucleotide sequence accession number. 

The full-genome sequence of MNSV Canadian isolate ABCA13-01 was deposited in GenBank under the accession number KR094068.
